# Association of Respiratory Syncytial Virus Toll-Like Receptor 3-Mediated Immune Response with COPD Exacerbation Frequency

**DOI:** 10.1007/s10753-017-0720-4

**Published:** 2017-12-21

**Authors:** Daishun Liu, Qian Chen, Honglan Zhu, Ling Gong, Yi Huang, Shiguang Li, Changwu Yue, Kaifeng Wu, Yang Wu, Wei Zhang, Guichuan Huang, Ling Zhang, Shenglan Pu, Daoxin Wang

**Affiliations:** 1 Department of Respiratory Medicine, the First People’s Hospital of Zunyi, the Third Affiliated Hospital of Zunyi Medical College, Institute of Respiratory Diseases in Zunyi, Zunyi, Guizhou 563002 China; 2 Department of Respiratory Medicine, The Second Affiliated Hospital of Chongqing Medical University, Chongqing, 400010 China

**Keywords:** TLR3, AECOPD, RSV, immune response

## Abstract

The objective of the study is to explore the role of respiratory syncytial virus Toll-like receptor 3 (TLR3)-mediated immune response in the pathogenesis of acute exacerbations of chronic obstructive pulmonary disease (AECOPD). A total of 20 AECOPD patients and 10 normal volunteers were studied. TLR3 was detected by RT-PCR, and respiratory syncytial virus (RSV) was detected by nested RT-PCR. Then, A549 cells were infected by RSV at different time points and at different viral titers. TLR3 mRNA was detected by RT-PCR, the protein of TLR3 and interferon regulatory factor 3 (IRF3) were detected by western blot, and IRF3 protein localization was detected by immunofluorescence. Interferon-β (IFN-β) and interleukin-6 (IL-6) were detected by ELISA. A total of 4 (20%) of the 20 AECOPD patients sampled were infected with RSV. The forced expiratory volume in 1 second (FEV_1_) percentage was lower in the AECOPD patients infected with RSV compared to those not infected (*P* = 0.03). The expression of IL-6 in the two groups was diametrically opposite (*P* = 0.04). The AECOPD group (*n* = 20) showed an increase in TLR3 mRNA compared with that of the control group (*n* = 10) (*P* = 0.02). The RSV-infected AECOPD group (*n* = 4) showed an obvious increase in TLR3 mRNA compared with that of the control group (*P* = 0.03). There was a significant correlation between severity of reduction in lung function at exacerbation and the increasing expression of TLR3 in AECOPD patients. The TLR3 signaling pathway was activated in lung epithelial cells. TLR3 mRNA/protein levels were increased in A549 infected with RSV compared with those of the control group. IRF3 protein also increased along with the occurrence of nuclear transfer in A549 infected with RSV. IFN-β and IL-6 were also increased in the RSV-infected A549 cells compared with those of the control (*P* = 0.00 and 0.00, respectively). Increased TLR3 expression in AECOPD patients is associated with declining lung function. TLR3 may be a risk factor for RSV-infected AECOPD patients.

## INTRODUCTION

Chronic obstructive pulmonary disease (COPD), a common preventable and treatable disease, is characterized by persistent airflow limitation that is usually progressive and associated with an enhanced chronic inflammatory response in the airways and lung to noxious particles or gases. Exacerbations and comorbidities contribute to the overall severity in individual patients. Currently, it is now one of the leading causes of mortality and morbidity worldwide and is an important contributor to the global burden of disease [1]. Although there has been some progress in the diagnosis and treatment of the disease, there is no special treatment for damage to lung function or systemic inflammation, and patient survival has not yet been extended.

Prior to the use of polymerase chain reaction (PCR)-based techniques for viral detection in acute exacerbation of chronic obstructive pulmonary disease (AECOPD) patient samples, approximately 50–70% of exacerbations were considered to be due to infection, 10% due to environmental agents, and 30% due to unknown etiologies. Isolated infectious agents were most often bacteria [2]. However, studies detecting viral infection using PCR methods have determined the incidence of virus-related AECOPD to be 56%; respiratory syncytial virus (RSV) infections make up a significant proportion of these [3–6]. COPD has been identified as an independent and significant risk factor for RSV infection that causes severe illness, hospitalization, and ICU admission [7]. Although the precise mechanisms of the onset of COPD exacerbations have not been fully clarified, the viral infection-mediated immune response is thought to play a role. To date, numerous studies have reported on Toll-like receptor 3 (TLR3) antiviral activity in *in vivo* and *in vitro* experiments, and related studies have shown that TLR3-mediated immune and inflammatory factors may play a pathogenic role in antiviral activities [8–14]. For example, in a study of TLR3-deficient mice infected with murine encephalomyelitis virus Taylor (TMEV), it has been suggested that TLR3 signaling may be either protective or pathogenic for the development of TMEV-induced demyelinating disease [9]. Furthermore, animal mortality was observed in other studies. TLR3-deficient mice appear to be more resistant to other infections compared to that of WT mice—they display enhanced resistance to influenza virus [10], Punta Toro virus [11], vaccinia virus [12], and West Nile virus (WNV) [13] infections. A weak inflammatory response in TLR3-deficient animals might contribute to the low disease severity in these mice. There have also been related studies in humans. For example, early herpes simplex virus-1 (HSV-1) infection suggests that human TLR3-dependent and interferon (IFN)-mediated immunity is essential for defense against HSV-1 in the central nervous system (CNS) during primary infection in childhood, but apparently otherwise largely redundant in host defense [14–16]. In studies of spleen-borne encephalitis by Kindberg [17] and Andrey V [18], it was suggested that a functional TLR3 is a risk factor for tick-borne encephalitis virus (TBEV) infection.

The above studies show that TLR3 may be a risk factor both in human and animal experiments. So, whether TLR3 is also a risk factor in AECOPD remains in question—Kinose D et al. has conducted a prospective observational study showing that TLR3 gene expression in sputum samples was not a significant predictor for COPD exacerbation [19]. RSV is the main pathogen COPD exacerbation; whether RSV-TLR3-mediated immune response plays an important role in the pathogenesis of COPD exacerbation needs to be explored. In our experiments, we detected RSV in sputum samples from patients with AECOPD. Then, we detected TLR3 in sputum samples from patients in the control group and the RSV-infected AECOPD group. Other causes of AECOPD and collected inflammatory factors, clinical signs, and lung function in the two groups were analyzed. Finally, TLR3-mediated inflammatory cytokine signaling pathways were confirmed in lung epithelial cells.

## MATERIALS AND METHODS

### Patient Selection

Patients with AECOPD and a group of normal patients were recruited from hospital clinics, outpatient clinics, and volunteers, between November 2012 and March 2013 in the First People’s Hospital of Zunyi, China. COPD was defined according to guidelines (Global Initiative for Chronic Obstructive Lung Disease, GOLD). Exacerbation was defined as increased dyspnea, cough, or sputum expectoration (quality or quantity) that led the subject to seek medical attention [20]. A clinician saw patients within 24 h to confirm the diagnosis [20] *via* medical history and physical examination and to perform blood gas analysis and administer oxygen as required. After initial treatment with inhaled bronchodilators, when clinical condition permitted, pulmonary function was assessed, and peripheral blood and sputum samples were obtained. All patients who had not received any antibiotics or systemic glucocorticoid therapy were enrolled. All patients at some stage of the study underwent high-resolution computed tomography (HRCT) except those with concomitant pneumonia, bronchiectasis, and/or tuberculosis. A clinician confirmed the control group *via* medical history and physical examination.

### Data Collection

The following parameters were recorded on admission: age, sex, smoking habits, current medication, clinical signs and symptoms of respiratory infection, pulmonary function testing, HRCT for identification of pulmonary infiltrates, interleukin-6 (IL-6), procalcitonin (PCT), blood gas, and routine blood chemistry and counts. Within 24 h of admission, sputum was collected; patients with less or without sputum were induced to produce sputum. TLR3 was detected in the sputum of AECOPD and control; RSV was detected in the sputum of AECOPD.

### Induced Sputum and Sputum Processing

Sputum was induced and sputum processing was performed according to previously published protocols [3].

### Materials

A549 lung epithelial cells (CCL-185™), HEp2 cells (CCL-23), and human RSV strain Long (VR-26) were obtained from the American Type Culture Collection (ATCC USA). Rabbit IgG anti-IRF3 (FL-425) and goat IgG anti-TLR3 (sc-8691) were obtained from Santa Cruz Biotechnology (USA). Monoclonal mouse IgG2a anti-β-actin (AA128-1) was obtained from Beyotime (China). Biotin-labeled goat anti-rabbit IgG, biotin-labeled donkey anti-goat IgG, and biotin-labeled goat anti-mouse IgG were obtained from Gene Company (China). The RNeasy Mini kit was obtained from Qiagen. Prime Script RT reagent Kit (real time), SYBR Premix Ex Taq™ II (real time), and Prime Script RT-PCR Kit were obtained from TaKaRa (Japan).

### Cell Culture and RSV Infection

A549 and HEp-2 cells were cultured in DMEM (Hyclone) supplemented with 10% fetal bovine serum (FBS; Hyclone), 100 U/mL of penicillin, and 25 mg/mL of gentamicin and were incubated at 37 °C and 5% CO_2_. RSV-infected A549 cells were maintained in DMEM supplemented with 2% FBS (maintenance medium) and were grown at 37 °C and 5% CO_2_.

### Preparation of RSV, Estimation of TCID50, and UV Inactivation

RSV was passaged in HEp-2 cells, which was grown in a maintenance medium and at 37 °C and 5% CO_2_. When the cytopathic effect reached 80–100%, the culture flasks were subjected to three freeze–thaw cycles and the supernatant was spun at low speed to eliminate cellular debris. The supernatant was aliquoted and frozen at − 80 °C until use. While a control group was established (maintenance medium instead of RSV), a culture medium of uninfected HEp-2 cells was collected in the same way, which was used as control in subsequent experiments—the TCID50 was determined using HEp-2 cells. Serial 10-fold dilutions were made of RSV stocks, and 50-μL samples of each dilution were added to duplicate wells of a 96-well plate containing a confluent monolayer of HEp-2 cells. Cytopathological assessment was performed after 10 days. The dilution causing cytopathic effects in half the cultures (the median tissue culture infective dose or TCID50) was then calculated as described by Reed and Muench (1938), and viral titers were expressed as TCID50 per unit volume of viral suspension [21]. UV inactivation (UV-RSV) was conducted in a Stratagene (Cedar Creek, TX) UV-stratalinker apparatus using 1800 mJ of UV radiation.

### RNA Extraction, Reverse Transcription, Real-Time PCR, and Semiquantitative RT-PCR

RNA extraction from sputum and cells was performed using a standard extraction kit (Qiagen RNeasy Mini kit). TLR3 complementary DNA (cDNA) preparation and real-time PCR were performed from sputum using the Prime Script RT reagent Kit (real time) and SYBR Premix Ex Taq™ II (real time), respectively. Quantitative PCR reactions were run on a light cycler real-time PCR system at 95 °C for 30 s, followed by 40 cycles of 95 °C for 5 s and 57 °C for 30 s. The melting program was 55 °C for 5 s, followed by 95 °C for 0.5 s. Table 1 shows that all primer sequences relative to levels of mRNA for each factor were normalized to β-actin—determined by using the Ct value and the formula transcription 2^−ΔΔ^Ct. RSV cDNA was prepared and nested PCR was performed from sputum using the Prime Script RT-PCR Kit. PCR reactions were run on a PCR system (BIO-RAD) at 94 °C for 30 s, followed by 30 cycles of 58 and 72 °C for 30 s. In the nested PCR step, 2 μL of the initial reaction product was added to a reaction mixture of 50 μL containing the same components as the first PCR step. Table 1 shows all primer sequences. Amplified PCR products were detected by electrophoresis on Goldview I-stained 2% agarose gels and photographed under UV illumination. RNA isolation and RT-PCR analysis were carried out by Rohde [3].

**Table 1:** Primer Sequences

Gene	Primer sequence 5′-3′ direction	Expected length of the PCR product (bp)
TLR3	GCAACAACAACA TAG CCAACA T (upper) GGA GGT GAG ACA GAC CCT TTA G (lower)	153
RSV [3]	CCA TTC TGG CAA TGA TAA TCT C GTT TTT TGT TTG GTA TTC TTT TGC AG CGG CAAACC ACAAAG TCA CAC GGG TAC AAA GTT AAA CAC TTC	326
β-actin	AGC GAG CAT CCC CCA AAG TT (upper) GGG CAC GAA GGC TCA TCA TT (lower)	285

TLR3 cDNA was prepared from cells and PCR was performed using the Prime Script™ RT-PCR Kit. PCR reactions were run on a PCR system (BIO-RAD) at 94 °C for 30 s, followed by 30 cycles of 57 and 72 °C for 30 s. Amplified PCR products were detected by electrophoresis on Goldview1-stained 2% agarose gels and photographed under UV illumination. A DNA size marker ladder (MW 50, 100, 150, 200, 300, 400, and 500 bp; Sangon Corp, Shanghai, China) was also used. The density of the bands was quantitated with the Labworks software imaging densitometer. Densitometry was expressed as fold increase (experimental value/b-actin value and experimental value/control value from three independent experiments).

### Western Blot Analysis of IRF-3 and TLR3

Cells were first washed in phosphate-buffered saline (PBS) and lysed in RIPA lysis buffer (Beyotime, China). The samples were left on ice for 30 min and centrifuged at 14,000*g* for 5 min; the supernatant containing total extracts was collected and assayed for TLR3 and IRF3 protein. Protein concentrations in lysates were determined using the BCA protein assay kit (Solarbio, China). A total of 20 μL of each sample containing 50 μg of protein was run on an 8% SDS, tris-glycine-polyacrylamide gel, and transferred to a PVDF membrane (Solarbio). The membrane was treated with a blocking buffer for 12 h at 4 °C, followed by incubation with rabbit IgG anti-IRF-3 and goat IgG anti-TLR3 at a 1:200 dilution in TBS containing 5% fat-free milk overnight at 4 °C. Subsequently, the membrane was incubated in a 1:2000 dilution of biotin-labeled goat anti-mouse IgG, biotin-labeled donkey anti-goat IgG, or biotin-labeled goat anti-rabbit IgG for 2 h at room temperature (RT). The membrane was washed three times, then scanned with an Odyssey (BIO-RAD) infrared imaging system, and densitometry of individual bands was performed with the Odyssey (BIO-RAD) imaging software. Densitometry was expressed as fold increase of experimental conditions compared with that of the control.

### Immunofluorescent Staining for IRF3

Cells grown on cover slips were fixed for 20 min with 4% fix and solubilized in PBS containing 0.2% Triton-X100 for 20 min at RT, followed by blocking with PBS containing 2% goat serum for 1 h at RT. Endogenous IRF3 was detected using a 1:50 dilution of SC-9082 followed by a 1:200 dilution of a goat anti-rabbit secondary antibody conjugated to FITC goat anti-rabbit IgG (Solarbio). DAPI (4′, 6-diamidino-2-phenylindole) was used as a nuclear counterstain. Samples were analyzed with a Nikon E80i epifluorescence microscope.

### ELISA for IFN-β and IL-6

IFN-β and IL-6 levels were determined using a standard ELISA kit (BLKW Biotechnology, China).

### Statistical Analysis

Baseline recruitment data are presented as medians (range). The remaining data are presented as the mean ± SE. Comparisons of two groups were made using analysis of *t* test. Comparisons of continuous variables among subgroups and multiple variables were made using analysis of variance (ANOVA). Correlation coefficients were calculated using the Pearson method. Significance was determined by SPSS19.0 statistical analysis software (Chicago, IL). A *P* value of < 0.05 was considered statistically significant.

## RESULTS

### Recruit Characteristics

A total of 40 AECOPD patients were enrolled in the study, 20 (50%) of whom could not enter the final analysis as a result of concurrent infections (pneumonia, *n* = 15; bronchiectasis, *n* = 3; tuberculosis, *n* = 4). Normal volunteers (*n* = 10) were recruited from outpatient clinics into the First People’s Hospital of Zunyi, China. Characteristics of the 30 participants are summarized in Table 2. There were no differences between the baseline characteristics in terms of sex distribution, current smoking status, or age between control and AECOPD groups.

**Table 2:** Basic Characteristics of Recruitment

Basic characteristics	Value
Control	
Recruitment numbers, *n*	10
Age, years (range)	61.5 (40–77)
Male/female, *n* (%)	6 (60), 4 (40)
Smoking habit	
Never, *n* (%)	4 (40)
Ex, *n* (%)	2 (20)
Current, *n* (%)	4 (20)
AECOPD	
Recruitment numbers, *n*	20
Age, years (range)	69.5 (49–85)
Male/female, *n* (%)	17 (85), 3 (15)
Smoking habit	
Never, *n* (%)	3 (15)
Ex, *n* (%)	8 (40)
Current, *n* (%)	9 (45)
GOLD severity class, *n* (%)	
1	1 (5)
2	10 (50)
3	8 (40)
4	1 (5)
Pulmonary function	
FVC, L (range)	1.65 (0.8–3.48)
FEV1, L (range)	1.08 (0.54–2.27)
FEV1% predicted, % (range)	51.06 (26.55–81.36)
FEV1/FVC, % (range)	64.99 (51.05–68.1)
Blood	
PO_2_, mmHg (range)	61.9 (48.9–90.7)
PCO_2_, mmHg (range)	39.85 (30.2–60.1)
WBC, 10^9^/L (range)	8.3 (4.5–14.8)
N% (range)	76.65 (57.9–92.8)
IL-6, pg/mL (range)	8.29 (1.5–50.7)
PCT, ng/mL (range)	0.057 (0.02–0.173)

### Detection of Respiratory Viruses and Correlation Between Virus Detection and Clinical Characteristics

**Fig. 1:**
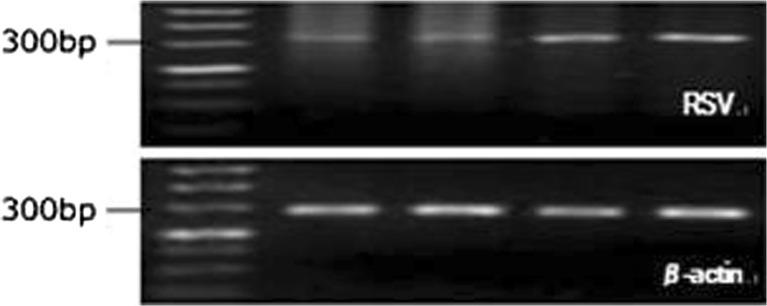
The presence of respiratory viruses in sputum of 20 AECOPD patients. The presence of RSV in all sputum samples of 20 AECOPD patients was assayed by nested polymerase chain reaction. Four of the 20 AECOPD patients sampled had detectable RSV.

Four of the 20 AECOPD patients sampled had detectable RSV (Fig. 1). RSV was detected in 20% of all sputum samples collected. Sequencing of RSV-positive PCR samples was confirmed by Rohde [3]. RSV was not detected in the control group. Table 3 shows the basic characteristics of the four AECOPD patients with detectable RSV and 16 AECOPD patients who did not have detectable RSV during the study. The AECOPD group with detectable RSV (*n* = 4) showed a decline in FEV1% (37.43 ± 9.89) and FEV1/forced vital capacity (FVC) (37.43 ± 9.89) compared with the FEV1% predicted (56.05 ± 15.1) and FEV1/FVC (63.94 ± 3.68) in the AECOPD group in which RSV was not detected (*n* = 16). The AECOPD group with detectable RSV (*n* = 4) showed an increase in IL-6 (26.26 ± 20.28) compared with that observed in the AECOPD group without RSV (*n* = 16) (9.43 ± 11.53). The differences in FEV1% predicted, decline in FEV1/FVC, and increase in IL-6 between these two groups were significant (*P* < 0.05; Table 3). There were no differences between the basic characteristics in terms of sex distribution, age, FVC, FEV1, PO_2_, POC_2_, WBC, N%, and PCT.

**Table 3:** The Basic Characteristics of RSV Were Detected Which Were Not Detected in Patients with AECOPD

Basic characteristics	RSVs were detected	RSVs were not detected	*P* value
Age, years	71.3 ± 9.32	69.2 ± 9.94	0.712
Pulmonary function			
FVC, L	1.44 ± 0.73	1.94 ± 0.77	0.271
FEV1, L	0.79 ± 0.35	1.25 ± 0.53	0.129
FEV1% predicted	37.43 ± 9.89	56.05 ± 15.1	0.037
FEV1/FVC %	56.91 ± 7.35	63.94 ± 3.68	0.014
Blood			
PO_2_, mmHg	56.05 ± 3.43	68.18 ± 11.57	0.064
PCO_2_, mmHg	42.1 ± 9.62	39.6 ± 6.09	0.563
WBC, 10^9^/L	8.38 ± 0.69	8.79 ± 3.26	0.46
N%	83.45 ± 3.87	73.74 ± 9.27	0.066
IL-6, pg/mL	26.26 ± 20.28	9.43 ± 11.53	0.041
PCT, ng/mL	0.09 ± 0.06	0.06 ± 0.04	0.363

### TLR3 Expression Increased in Sputum of AECOPD

**Fig. 2:**
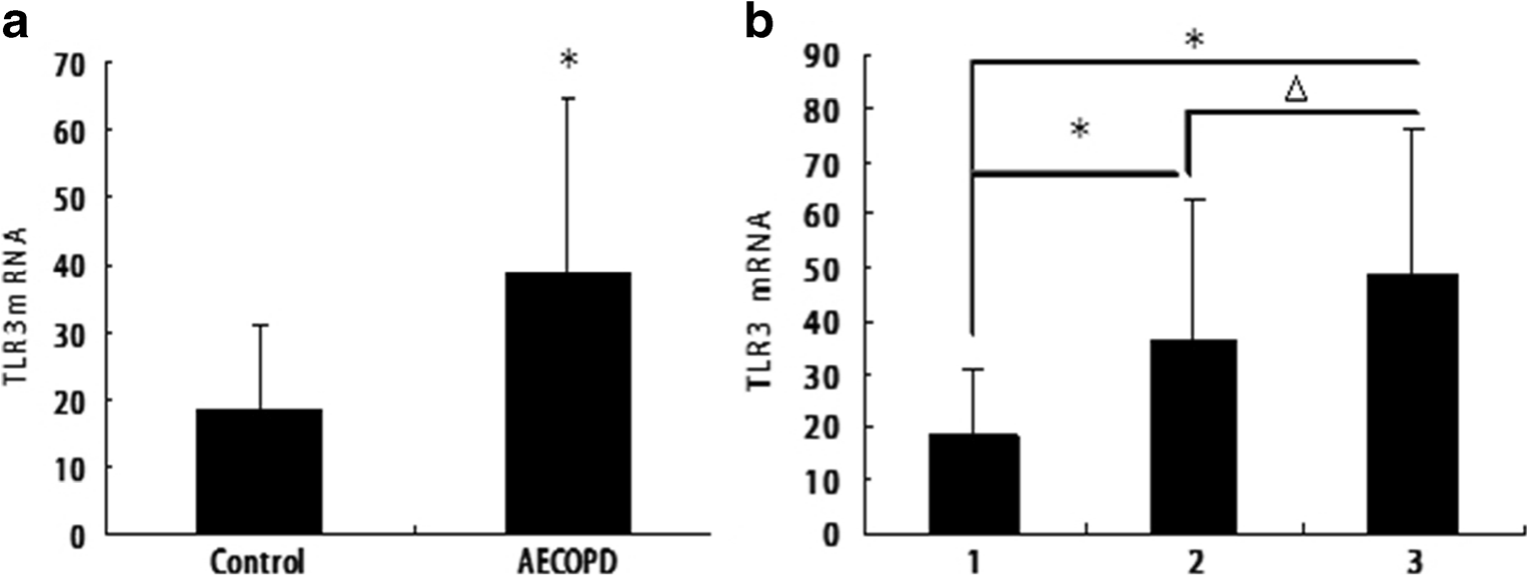
TLR3 expression increased in sputum of AECOPD patients. **a** The AECOPD group showed an increase in TLR3 mRNA expression compared with that in the control (*n* = 10). The difference between these two groups was significant (*P < 0.05). Data represent the mean ± SE from 10 normal recruits and 20 AECOPD patients by real-time PCR. **b** Both the RSV-positive AECOPD (*n* = 4) and RSV-negative AECOPD groups (*n* = 16) showed an increase in TLR3 mRNA compared with that of the control; the difference between these two groups was significant (*P < 0.05). The RSV-positive AECOPD group showed an increase in TLR3 mRNA compared with that of the RSV-negative AECOPD group; the difference between these two groups was not significant (△*P* > 0.05), as indicated (1, 2, 3 = control, AECOPD without RSV, AECOPD with RSV, respectively). Data represent the mean ± SE from 10 normal subjects, 16 AECOPD subjects without RSV, and 4 AECOPD subjects with RSV by real-time PCR, respectively.

TLR3 mRNA was detected in sputum samples from all 30 recruits by real-time PCR. The AECOPD group (*n* = 20) showed an increase in TLR3 mRNA (38.72 ± 26.25), compared with that in the control (*n* = 10; 18.35 ± 12.74). The difference in TLR3 mRNA increase between these two groups was significant (*P* < 0.05; Fig. 2a). The AECOPD group with RSV (*n* = 4) showed an increase in TLR3 mRNA (48.66 ± 27.64) compared with that in the control group (*n* = 10; 18.35 ± 12.74). The AECOPD group without RSV (*n* = 16) also showed an increase in TLR3 mRNA (36.24 ± 26.21) compared with that in the control group (*n* = 10; 18.35 ± 12.74). The difference in TLR3 mRNA increase between these two groups was also significant (*P* < 0.05; Fig. 2b). The AECOPD group with RSV (*n* = 4) also showed an increase in TLR3 mRNA (48.66 ± 27.64) compared with that in the AECOPD group without RSV (*n* = 16; 36.24 ± 26.21). The difference in TLR3 mRNA increases between these two groups was not significant (*P* > 0.05 Fig. 2b).

### Increased Severity of AECOPD Associated with TLR3

**Fig. 3:**
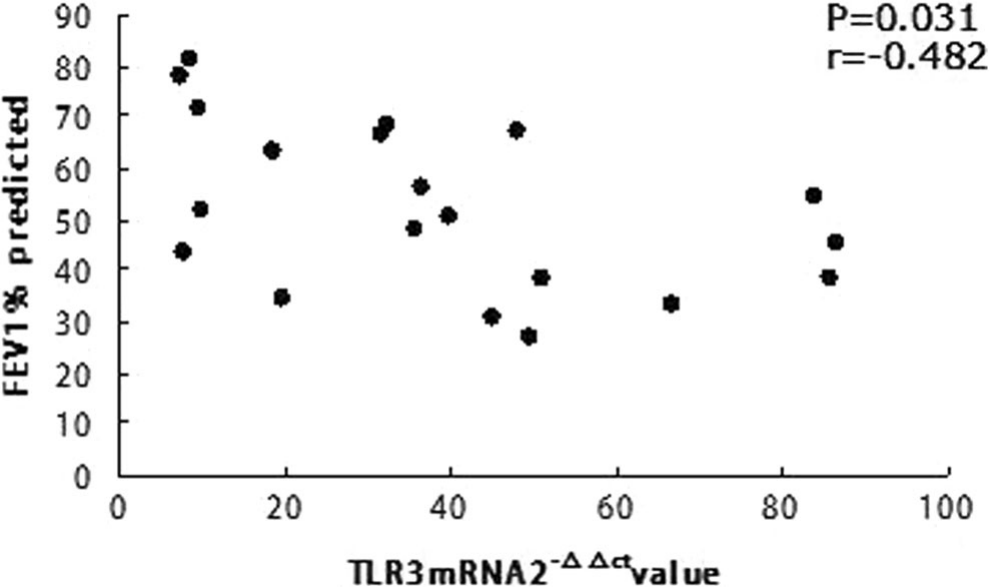
Increased severity of exacerbations associated with TLR3: relationship between an increase in sputum TLR3 during severe COPD exacerbations and severity of exacerbation expressed as percentage reduction in lung function (% predicted FEV1) at exacerbation. Data from all 20 patients.

Sputum TLR3 mRNA was markedly increased during exacerbations (Fig. 2a) in the 20 AECOPD patients. A significant relationship between TLR3 and exacerbation severity was demonstrated by significant correlations between severity of reduction in lung function (% of predicted FEV1) at exacerbation and increase in sputum TLR3 (Fig. 3; *r* = 0.482; *P* = 0.031).

### Increased TLR3 Expression After RSV Infection in A549 Cells

**Fig. 4:**
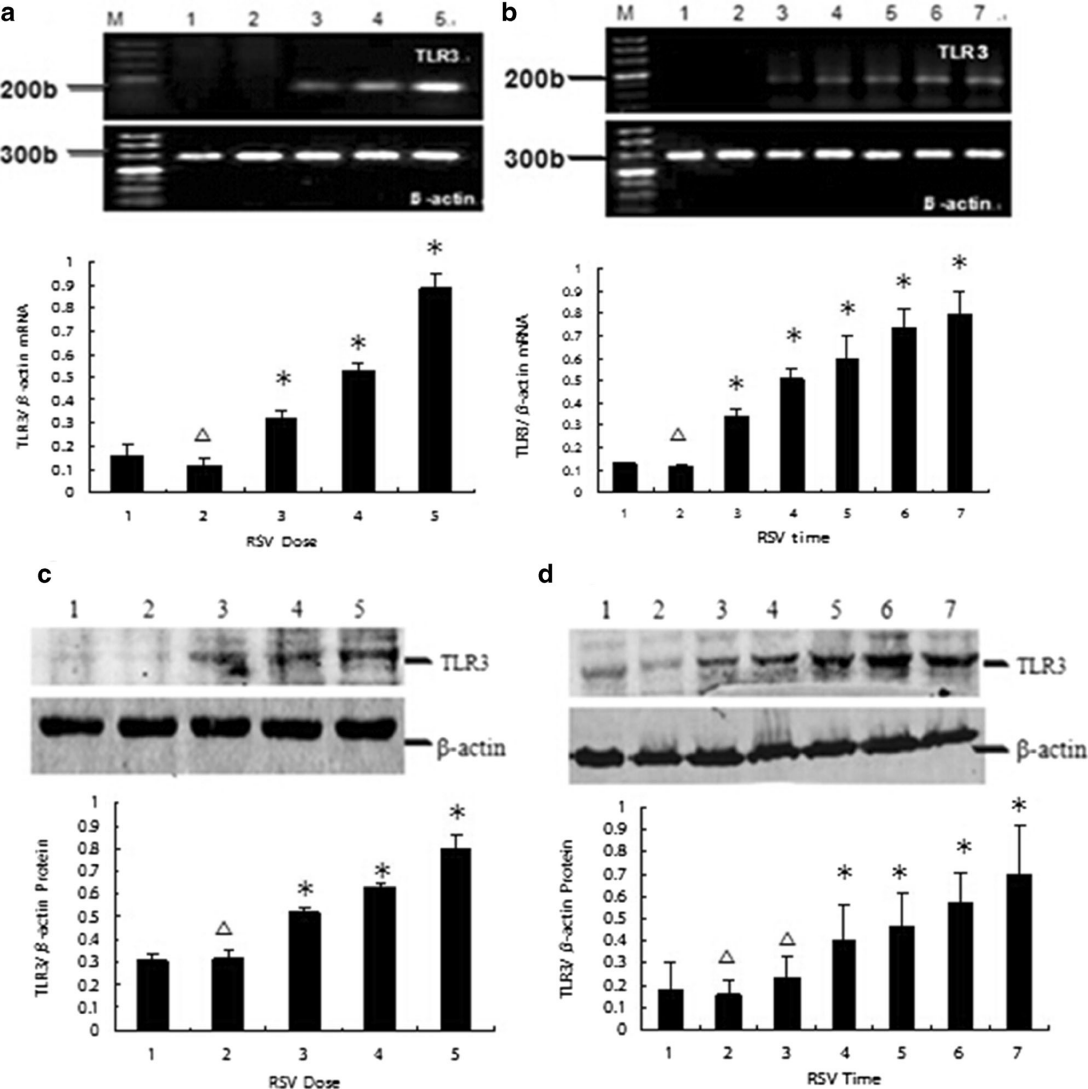
Increased TLR3 mRNA/protein expression after RSV infection in A549 cells. **a**, **c** TLR3 mRNA/protein in an RSV-dose-dependent manner in A549 cells was cultured for 36 h in the presence of medium, 10^3^UV-inactivated, 10^2^TCID50, 5 × 10^2^TCID50, and 10^3^TCID50 of infectious RSV particles, respectively, as indicated (1, 2, 3, 4, and 5, respectively). **b**, **d** TLR3 mRNA/protein in an RSV time-dependent manner in A549 cells was cultured for 6, 12, 18, 24, and 36 h in the presence of 10^3^TCID50 of infectious RSV particles, respectively, and cultured for 36 h in the presence medium or 10^3^TCID50 UV-inactivated RSV, as indicated (1, control; 2, UV-RSV; 3–7, RSV infection at 6, 12, 18, 24, and 36 h, respectively). TLR3 mRNA/protein expression was determined by reverse transcription-polymerase chain reaction (RT-PCR) and western blot analysis, respectively. Chart data represent the mean ± SE from three times per point. The relative level of target gene expression was determined by using Labworks software *P < 0.05 compared with control; △*P* > 0.05 compared with control.

The ability of A549 cells to express the *TLR3* gene was determined by semiquantitative RT-PCR analysis of total cellular RNA. Total RNA isolated from A549 cells was reverse transcribed and amplified with the specific primers described above. A549 cells were incubated with medium or RSV-titrated HEp-2 cell tissue culture supernatant containing various amounts of infectious RSV particles (UV-inactivated 10^3^TCID50, 10^2^TCID50, 5 × 10^2^TCID50, and 10^3^TCID50) and were cultured for 36 h. The culture medium of uninfected HEp-2 cells was used as a control. Figure 4a shows the *TLR3* gene in an RSV-dose-dependent manner. The most obvious expression of *TLR3* was observed with 10^3^TCID50 of infectious RSV particles compared with that of the control in this study. Compared with the control group, there was no significant increase in TLR3 mRNA expression in the UV-RSV group. A549 cells were also cultured for 6, 12, 18, 24, and 36 h in the presence of 10^3^TCID50 of infectious RSV particles, as well as for 36 h in the presence of the medium of 10^3^TCID50 of UV-inactivated infectious RSV particles. Figure 4b shows the expression of the *TLR3* gene in an RSV time-dependent manner. The most obvious expression of the *TLR3* was observed after 36 h of incubation compared with that of the control. Compared with the control group, there was no significant increase in *TLR3* gene expression in the UV-RSV group.

TLR3 protein level was determined by western blot analysis performed using the method described above. Equal protein loading was confirmed by examining β-actin protein expression. The current study shows that RSV infection increases TLR3 protein expression in A549 cells in a time- and RSV-dose-dependent manner (Fig. 4c, d). The most obvious expression of the TLR3 protein was observed after 36 h in the presence of 10^3^TCID50 of infectious RSV particles compared with that of the control. However, the expression of the TLR3 protein at 12 h was significantly higher than the expression of the *TLR3* gene at 6 h. Compared with the control group, there was no significant increase in TLR3 protein expression in the UV-RSV group.

### Increased IRF3 Expression and Nuclear Translocation After RSV Infection in A549 Cells

**Fig. 5:**
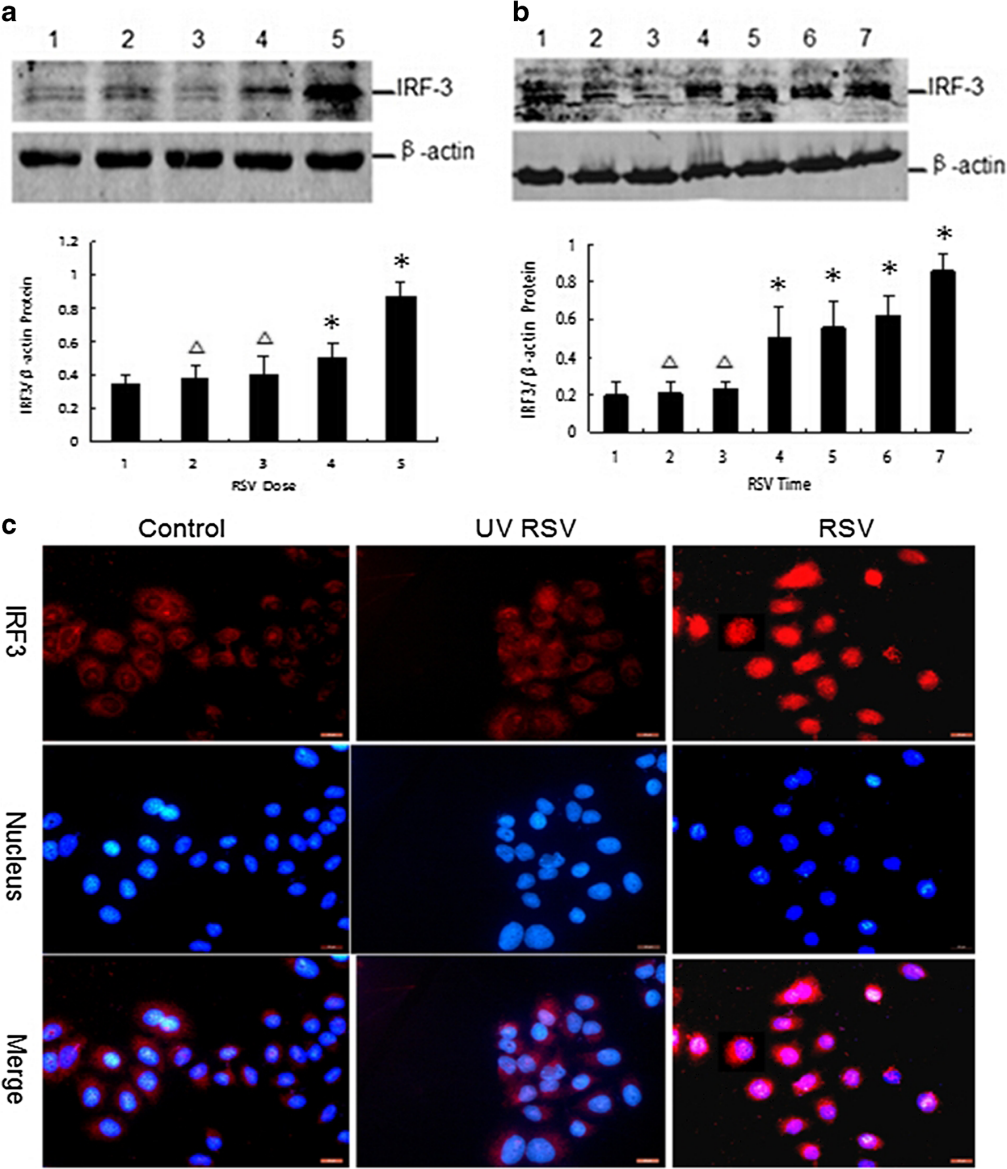
Increased IRF3 expression and nuclear translocation after RSV infection in A549 cells. **a** RSV-dose-dependent IRF3 protein expression in A549 cells cultured for 36 h in the presence of medium, 10^3^TCID50, UV-inactivated, 5 × 10^2^TCID50, and 10^3^TCID50 of infectious RSV particles as indicated (1, control; 2, UV-RSV; and 3, 4, 5, RSV 10^2^TCID50, 5 × 10^2^TCID50, 10^3^TCID50, respectively). **b** RSV time-dependent IRF3 protein expression in A549 cells cultured for 6, 12, 18, 24, and 36 h in the presence of 10^3^TCID50 of infectious RSV particles, and 36 h in the presence of medium or 10^3^TCID50 UV-inactivated of RSV, as indicated (1, control; 2, UV-RSV; 3–7, RSV infection 6, 12, 18, 24, and 36 h, respectively). IRF3 protein expression was determined by western blot analysis. Chart data represent the mean ± SE from three times per point. The relative level of target gene expression was determined using Labworks software (*P < 0.05 compared with control; △*P* > 0.05 compared with control). **c** Nuclear translocation of IRF3: A549 cell were stimulated with medium, 10^3^TCID50 UV-RSV, or 10^3^TCID50 of infectious RSV particles and cells were methanol-fixed at 36 h and stained for IRF3 (labeled red) to detect nuclear translocation. DAPI (blue) served as a nuclear counterstain.

IRF3 protein level was determined by western blot analysis using the method described above. Equal protein loading was confirmed by examining β-actin protein expression. A549 cells were incubated with medium or RSV-titrated HEp-2 cell tissue culture supernatant containing various amounts of infectious RSV particles (UV-inactivated 10^3^TCID50, 5 × 10^2^TCID50, 10^3^TCID50) and cultured for 36 h. The culture medium of uninfected HEp-2 cells was used as a control. Although there was not a significant increase in IRF3 protein expression in the 10^2^TCID50 of RSV, Fig. 5a shows the expression of IRF3 protein in an RSV-dose-dependent manner. The most obvious expression of protein was still observed with 10^3^TCID50 of infectious RSV particles compared with that of the control. Compared with the control group, there was no significant increase in IRF3 protein expression in the UV-RSV group. A549 cells were also cultured for 6, 12, 18, 24, and 36 h in the presence of 10^3^TCID50 of infectious RSV particles and 36 h in the presence of medium or 10^3^TCID50 of UV-inactivated infectious RSV particles. Figure 5b shows the expression of IRF3 in an RSV time-dependent manner. As was observed for the expression of TLR3 protein, the most obvious expression of IRF3 was observed after 36 h of incubation time and expression was observed after 12 h of incubation time compared with that of the control. Compared with that of the control group, there was no significant increase in IRF3 protein expression in the UV-RSV group.

Next, immunofluorescence was carried out to investigate protein location of IRF3 (Fig. 5c). A549 cells were cultured for 36 h in the presence of medium, 10^3^TCID50UV-RSV, or 10^3^TCID50 of infectious RSV particles. IRF3 was localized exclusively to the cytoplasm. RSV infection induced nuclear translocation of IRF3 in A549 cells.

### RSV Induced IFN-β and IL-6 Protein Expression in A549 Cells

**Fig. 6:**
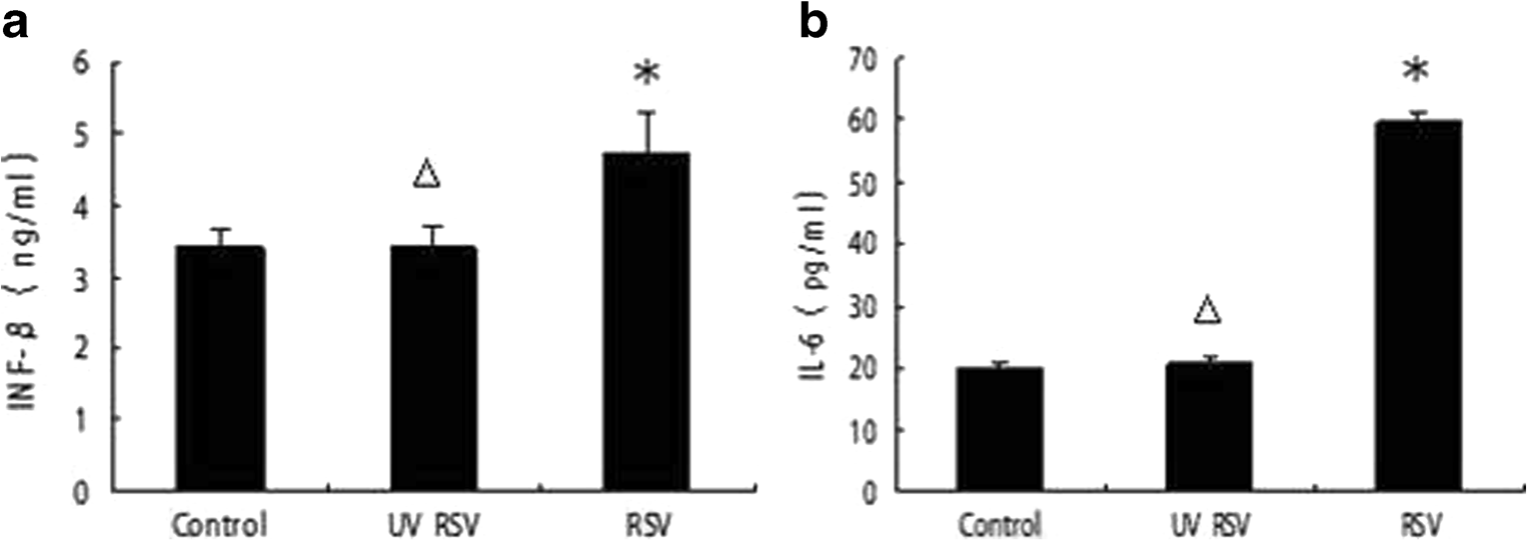
RSV induced IFN-β and IL-6 protein expression in A549 cells. A549 cells were stimulated with medium, 10^3^TCID50 UV-RSV, or 10^3^TCID50 of infectious RSV particles, and IFN-β (**a**) and IL-6 (**b**) concentrations in cell culture supernatants at 36 h post infection were measured by ELISA. Data represent the mean ± SE from three times (*P < 0.05 compared with control, △*P* > 0.05 compared with control).

A549 cells were stimulated with medium, 10^3^TCID_50_ UV-RSV, or 10^3^TCID_50_ of infectious RSV particles, and IL-6 and IFN-β concentrations in cell culture supernatants were measured by ELISA 36 h post infection. The RSV group showed an increase in IFN-β (4.74 ± 0.56) compared with that in the control group (3.40 ± 0.29); the difference in IFN-β increase was significant (*P* < 0.05; Fig. 6a). Similar to that observed for IFN-β expression, the RSV group also showed an increase in IL-6 (59.65 ± 1.64) compared with that in the control (19.87 ± 0.88); the difference in IL-6 increase was also significant (*P* < 0.05; Fig. 6b). However, in the above two experiments, the UV-RSV group did not show an increase in IFN-β or IL-6 compared to that of the control.

## DISCUSSION

This is the first study to investigate the role that TLR3 may play in the etiology and progression of AECOPD. We show that TLR3 mRNA can be detected in the sputum of many patients with AECOPD, and its detection may be associated with a decline in lung function.

TLR3 is one of the TLRs and is a pattern-recognition receptor. TLR3-expressing cells include dendritic [22], CD8+ T [23], NK [24], retinal [25], corneal [26], and intrahepatic biliary cells [27], as well as intestinal [28] epithelial cells, keratinocytes [29], lung and dermal fibroblasts [30, 31], vessel endothelial cells [32], hepatocytes [33], and CNS-resident cells, including neurons, oligodendrocytes, astrocytes, and microglia [34, 35]. A total of 13 kinds of TLRs have been found in humans and mice *in vivo*. TLRs recognize pathogen-associated molecular patterns expressed by infectious agents and mediate production of antimicrobial peptides and cytokines needed for host defense. TLRs 1, 2, 6, and 10 recognize bacterial lipoproteins; TLRs 4 and 5 recognize LPS and flagellin, respectively; TLRs 7, 8, and 9 recognize nucleic acid molecules; TLRs 10, 11, and 12 recognize actin-like molecules; and TLR3 recognizes dsRNA, an intermediate generated during most viral infections.

In this study, 4 of the 20 (20%) AECOPD patients sampled had RSV detected in their sputum. A lower incidence of RSV (10.5%) was observed in the sputum of AECOPD patients by Rohde [3], while higher incidences (32.8 and 28%) were observed in AECOPD patients by Tom [36] and Borg [37], respectively. Variation in the incidence of RSV infection among the different studies is likely attributable to differences in study populations, seasonal and regional variation, sample acquisition and type, and PCR assay systems.

RSV is an established cause of acute respiratory illness in children, and RSV bronchiolitis is associated with the development of persistent wheeze in later childhood [38]. Tom [36] shows that RSV detection was associated with a decline in FEV1% predicted and heightened airway inflammation in terms of increased levels of IL-6 and IL-8. In the same study, Tom also showed that an RSV infection may persist in certain populations. We show that RSV detection is associated with a decline in FEV1% predicted (Table 2) and higher levels of airway inflammation marker, IL-6 (Table 2). These studies suggest that RSV may play a role in the pathogenesis of airway inflammation and subsequent deterioration in lung function in the COPD. RSV may have proinflammatory effects; it is also possible that it acts by modulating the response of lung cells to other inflammatory stimuli, including bacterial lipopolysaccharide [39], or by promoting neutrophil adhesion, thereby augmenting lung damage [40].

Currently, TLR3 research focuses on its antiviral activity, and both human and animal studies suggest that TLR3 may be a risk factor for viral infection. Studies that detect viral infection using PCR-based methods have determined the incidence of virus-related AECOPD to be 56%, which also contributed to our study of the TLR3 in the AECOPD. Our data show higher levels of TLR3 mRNA in sputum samples of patients with AECOPD than those of controls by real-time PCR (Fig. 3a; *P* < 0.05). However, no difference was observed between the RSV-positive AECOPD group and the RSV-negative AECOPD group with regard to the levels of TLR3 mRNA in sputum samples (Fig. 3a; *P* > 0.05). This may be due to the presence of other viral or bacterial infections in AECOPD patients—after all, RSV may contribute to only a small portion of the etiology of AECOPD.

We found a significant relationship between TLR3 and exacerbation severity, demonstrated by significant correlations between severity of reduction in lung function (FEV1% predicted) at exacerbation and increase in sputum TLR3 (Fig. 3; *r* = 0.482, *P* = 0.031). However, we did not observe TLR3 to be associated with IL-6, PCT, WBC, N%, PO_2_, PCO_2_, FVC, or FEV1 in AECOPD subjects. This may be unexpected, but it might prompt other clinical studies, such as the spleen-borne encephalitis. TLR3 may be a risk factor in acute exacerbation of COPD, having been challenged by viruses.

TLR3 consists of an extracellular leucine-rich repeat (LRR) motif, a transmembrane (TM) domain, and an intracellular Toll and IL-1R (TIR) domain [41]. TLR3 signaling will transduce down, depending on these three domains: the leucine-rich repeat responsible for recognizing PAMPs, and the transmembrane (TM) and intracellular Toll and IL-1R (TIR) domains responsible for down transduction of the activation signal [41]. The TLR3 signaling pathway is mediated exclusively by the TRIF adapter [42], which is recruited to TLR3 by interaction between the TIR domains of the two molecules. Various branches of the signaling pathway emanating from TLR3–TRIF lead to the activation of IRF3 and NF-kB [43]. This pathway together induces the production of antiviral IFNs and other cytokines [44]. We sought to determine whether the TLR3-mediated immune response also works *via* this pathway in the lung epithelial cells. Thus, we conducted an experimental study in lung epithelial cells.

In this study, we demonstrate that RSV increases the expression of TLR3 on the surface of airway epithelial cells (Fig. 4). Then, we observed increased IRF3 expression and nuclear translocation after RSV infection in our study (Fig. 5). We know that activation of the IRF3 pathway results in expression of type I interferon including the IFN-α and IFN-β. The RSV infection group also showed an increase in IFN-β compared with that of the control and UV-RSV groups by ELISA (Fig. 6a). We know activation of NF-kB pathways results in expression of various inflammatory mediators, including the cytokines TNF-α and IL-6 and the chemokine IL-8. A TLR3-NF-kB pathway of airway epithelial cells was detected in the study of Dayna [45] and their study showed increased IL-8 mRNA and protein was accompanied by increased NF-kB nuclear localization. We also detected NF-kB-related inflammatory cytokine IL-6 and our data showed that IL-6 protein increased after RSV infection of airway epithelial cells. These studies demonstrate that RSV induces increased TLR3, IRF3, and NF-kB in airway epithelial cells, priming them for an enhanced inflammatory response when RSV induces their antiviral properties. These observations suggest that TLR3 might be an important target for therapy in RSV infection.

In conclusion, we have shown that TLR3 RNA can be detected from lower airway samples of patients with AECOPD. This is the first detection of TLR3 RNA in sputum of AECOPD patients. It is unclear what kind of role of TLR3 has in the pathogenesis of AECOPD. Our data showed TLR3 RNA detection was associated with FEV1% predicted in these AECOPD patients. The results of this study show that TLR3 may play a risk role in AECOPD patients, possibly due to viral and bacterial infection induced TLR3 activation. TLR3 also enhanced the inflammatory response when in an antiviral state, thereby augmenting lung damage. TLR3 was not associated with inflammatory cytokines (including IL-6, PCT, WBC, and N%) in our study. The reason for the discrepancy from findings of previous studies may be due to differences in detection method, different seasons, different time of sample collection, and other inflammatory markers not detected (*e.g.*, IL-8, TNF). At the same time, we did not carry out research or analysis in patients with stable-state COPD. Therefore, further studies with more clinical trials, more sophisticated designs, and more patients/controls are needed.
